# A Comparison of Metacognitive Therapy in Current Versus Persistent Depressive Disorder – A Pilot Outpatient Study

**DOI:** 10.3389/fpsyg.2019.01714

**Published:** 2019-08-06

**Authors:** Lotta Winter, Julia Gottschalk, Janina Nielsen, Adrian Wells, Ulrich Schweiger, Kai G. Kahl

**Affiliations:** ^1^Department of Psychiatry, Social Psychiatry and Psychotherapy, Hannover Medical School, Hannover, Germany; ^2^Division of Psychology and Mental Health, University of Manchester, Manchester, United Kingdom; ^3^Greater Manchester Mental Health NHS Trust, Manchester, United Kingdom; ^4^Department of Psychiatry and Psychotherapy, University of Lübeck, Lübeck, Germany

**Keywords:** metacognitive therapy, MCT, major depressive disorder, persistent depressive disorder, thinking style, metacognition, psychotherapy

## Abstract

**Background:** Metacognitive therapy (MCT) is a modern approach with demonstrated efficacy in current major depressive disorder (MDD). The treatment aims to modify thinking styles of rumination and worry and their underlying metacognitions, which have been shown to be involved in the initiation and perpetuation of MDD. We hypothesized that metacognitive therapy may also be effective in treating persistent depressive disorder (PDD).

**Methods:** Thirty depressed patients (15 with MDD; 15 with PDD) were included. Patients in both groups were comparable on depression severity and sociodemographic characteristics, but PDD was associated with more former treatments. Metacognitive therapy was applied by trained psychotherapists for a mean of 16 weeks.

**Results:** We observed a significant improvement of depressive symptoms in both groups, and comparable remission rates at the end of treatment and after 6 months follow-up. Furthermore, we observed significant and similar levels of improvement in rumination, dysfunctional metacognitions, and anxiety symptoms in both groups.

**Limitations:** The study is limited by the small sample size and a missing independent control group. The effect of the therapeutic alliance was not controlled. The quality of depression rating could have been higher.

**Conclusions:** We demonstrated that metacognitive therapy can successfully be applied to patients with PDD. The observed results were comparable to those obtained for patients with current major depressive disorder. Further studies with larger groups and a randomized design are needed to confirm these promising initial findings.

## Introduction

Major depressive disorder (MDD) is a debilitating and often treatment-refractory mental health problem and a significant cause of lost life years (Whiteford et al., 2013). This has led to a call for a continuous process of innovation in the field of depression treatment (Hollon et al., 2014; Riihimaki et al., 2014). Comorbidity and chronicity are common in MDD and frequently complicate the course of disease (Penninx et al., 2011).

To date, little is known about the underlying reasons for chronic disease courses, and how to manage them. Former studies pointed to the role of previous episodes and subclinical symptoms as course modifiers in MDD, leading to the development of chronicity (Hardeveld et al., 2010, 2013; Seemuller et al., 2014). Furthermore, maladaptive cognitive processes such as rumination and worrying have been shown to negatively influence the course of depression (Nolen-Hoeksema et al., 2008; Lyubomirsky et al., 2015). Rumination is defined as a negative pattern of responding to distress by repetitively focusing on the meanings, causes, and consequences of one’s depressive symptoms and has been linked with symptom severity and a chronic disease course in MDD (Kuehner and Weber, 1999; Wiersma et al., 2011; Gan et al., 2015). Klein (2010) summarized earlier onset, higher comorbidity rate, more extreme personality traits, higher levels of at least some cognitive biases, and greater suicidality as differences between chronic and non-chronic depression.

For the treatment of chronic depression several factors to consider when choosing treatment have been suggested (Kriston et al., 2014). A conclusion to date is that chronic depressions appear to require somewhat different approaches to treatment than non-chronic depressions (Klein, 2010).

In the following study, we aimed to examine the effects of a modern and effective form of psychological treatment, metacognitive therapy (MCT; Wells, 2000), in patients with current MDD and persistent depressive disorder (PDD). MCT differs significantly from other forms of psychotherapy in its focus on metacognitive processes and metacognitive beliefs as well as on regulating thinking styles, in contrast to traditional cognitive therapy where cognitive content is the target of psychotherapeutic intervention. MCT is based on the Self-Regulatory Executive Function model (Wells and Matthews, 1994) in which psychological disorder is caused and maintained by a transdiagnostic process of extended negative thinking and coping behaviors that lead to failures of effective self-regulation. The treatment focuses on changing cognitive processes and facilitating metacognitive modes of processing which can overcome inflexibility of attentional control that contributes to sustained repetitive negative thinking of rumination, worrying, and threat-monitoring. It is assumed that these thinking styles are also maintained by metacognitive beliefs, the latter are considered as a common factor in psychopathology leading to exacerbation of negative affect. In the metacognitive model of depression, positive metacognitive beliefs about the value of rumination in solving problems and overcoming low mood are thought to commonly occur. However, negative metacognitive beliefs concerning the uncontrollability of rumination and depressive thinking are considered central. The typical patterns of repetitive negative thinking in depression consist of rumination, threat-monitoring, and dysfunctional coping strategies (Wells, 2009). Consistent with this model, repetitive negative thinking is a transdiagnostic factor (Drost et al., 2014), rumination is an independent contributor to the maintenance of depressive symptomatology (Lyubomirsky et al., 2015; Yilmaz et al., 2015), and metacognitive beliefs contribute to depressive symptoms and rumination (Papageorgiou and Wells, 2003).

Some preliminary studies have examined the effects associated with MCT in current, recurrent, and postpartum MDD (Bevan et al., 2013; Callesen et al., 2014; Farahmand et al., 2014; Jordan et al., 2014; Normann et al., 2014; Dammen et al., 2015; Hagen et al., 2017). In each study, MCT was associated with large effect sizes and high levels of remission (Normann and Morina, 2018). So far, two studies have examined purely treatment-refractory cases (Wells et al., 2012; Papageorgiou and Wells, 2015), but no study to date has directly compared outcomes in current MDD versus PDD. As the effect sizes found in these studies were similar to non-refractory depression, the primary hypothesis of our pilot study was that MCT should have similar effectiveness in treating current MDD and PDD. We also predicted that the underlying maladaptive thinking style will change with treatment response in both subgroups.

## Materials and Methods

The study was approved by the local ethics committee (No. 1343-2012). Participants were consecutively recruited from a waiting list of the psychotherapy outpatient clinic of the Department of Psychiatry, Social Psychiatry and Psychotherapy of the Hannover Medical School. They were either referred by local psychiatrists, local general practitioners, or from other departments of the Hannover Medical School. All patients gave written informed consent to participate in the study.

### Participants

Inclusion criteria were a diagnosis of current MDD or PDD according to DSM-IV and an age between 18 and 70 years. Exclusion criteria were: cognitive impairment, current substance use disorder, psychotic disorder, bipolar disorder, acute medical conditions such as cancer or heart failure and suicidality requiring inpatient treatment.

Fifty patients were contacted for a first screening. Of these, 20 were excluded as they did not meet study criteria or were not willing to participate. Thirty patients gave informed consent. Of these, according to DSM-IV, 15 patients were diagnosed as having current MDD and 15 patients were diagnosed with PDD. Depressive symptoms had lasted for at least 2 years in patients with PDD. Furthermore, all patients with PDD had former treatment with antidepressant medication, and 11 PDD patients had at least one trial of cognitive behavioral therapy (CBT) without response (Table 1). In summary, PDD patients suffered from persistent and treatment-resistant depressive symptoms.

**Table 1:** Baseline demographics and sample characteristics of the patients (intention to treat, *n* = 30).

	MDD (*n* = 15)	PDD (*n* = 15)	*p*
Age (years)	42.9 (±13.2)	39.3 (±8.4)	0.4
Female (n/%)	10 (66.6%)	8 (53.3%)	0.4
Partnered (n/%)	8 (53.5%)	8 (53.3%)	0.6
Working (n/%)	14 (93.3%)	12 (80%)	0.3
Psychotherapy (n/%)	0 (0%)	11 (73.3%)	0.01
Pharmacotherapy (n/%)	0 (0%)	15 (100%)	0.00
Any comorbid psychiatric disorder (n/%)	10 (66.7%)	8 (53.3%)	0.4
Any anxiety disorder (n/%) Panic disorder (n/%) Social phobia (n/%) GAD (n/%) OCD (n/%) PTSD (n/%) Somatization disorder (n/%) Other anxiety disorder (n/%) Any personality disorder (n/%) Paranoid PD (n/%) Emotionally unstable PD (n/%) Avoidant PD (n/%) Narcissistic PD (n/%) Combined PD (n/%) Other (ADHD) (n/%)	6 (40%) – 1 (6.7%) 1 (6.7%) 1 (6.7%) – 2 (13.3%) 1 (6.7%) 3 (20%) – 2 (20%) 1 (6.7%) – – 1 (6.7%)	2 (13.3%) 1 (6.7%) – – – 1 (6.7%) – – 6 (40%) 1 (6.7%) 2 (20%) – 1 (6.7%) 2 (20%) –	0.3

*MDD, Current major depressive disorder; PDD, Persistent depressive disorder; GAD, Generalized anxiety disorder; OCD, Obsessive-compulsive disorder; PTBS, Post-traumatic stress disorder; PD, Personality disorder; ADHD, Attention-deficit hyperactivity disorder*.

For all patients who were on antidepressant medication when entering the study, two criteria were mandatory: they had to be on the current dose for at least 3 months before starting with MCT and they had to agree not to change the medication or dose until the end of therapy. A total of 10/15 patients with MDD and 12/15 patients with PDD reported former depressive episodes.

Of all, 27 patients reached the post-treatment evaluation, and 20 patients reached the 6 months follow-up. Baseline pre-treatment data are given in Table 1.

### Assessment and Design

A comprehensive pre-treatment assessment included a mental status examination, a semi-structured interview to document sociodemographic information and to screen the available psychiatric and medical information for the presence or absence of exclusion and inclusion criteria. Comorbidity status was assessed using a standardized diagnostic interview (SCID-1/SCID-2) (Wittchen et al., 1997). Depression severity was assessed using the German version of the clinician-rated 21-item Hamilton Depression Scale (Ham-D) (CIPS – Collegium Internationale Psychiatriae Scalarum, 2015). The severity of anxiety symptoms was assessed with the German version of the Beck Anxiety Inventory (BAI) (Kabacoff et al., 1997). Problematic metacognitive processes were evaluated using German versions of the Positive Beliefs about Rumination Scale (PBRS) (Papageorgiou and Wells, 2001b), the Negative Beliefs about Rumination Scale (NBRS) (Papageorgiou and Wells, 2001a), the short form of the metacognitions questionnaire MCQ-30 (Wells and Cartwright-Hatton, 2004), and the ruminative response scale (RRS) of the response styles questionnaire (Nolen-Hoeksema and Morrow, 1991).

Before therapy started, a preparatory session was used to give feedback on the diagnoses assessed and to set the patient’s personal therapy goals. The treatment for depression followed the treatment manual by Wells (2009) which is available in German (Wells, 2011). Therapy was terminated by agreement between therapist and patient when subjective therapy goals were met (T1). Mean treatment duration was 16 weeks (±8) with a frequency of one session per week in both groups. Six months after the end of therapy, patients were contacted and assessed for follow-up (T2). Twenty patients reached the 6-month follow-up examination (T2).

The main outcome criterion was improvement of depressive symptoms evaluated with the HamD. Secondary outcome parameters were changes in BAI and the evaluation of the PBRS, NBRS, MCQ-30, and the RRS.

A complete set of questionnaires and interviewer-rated measures was administered at pre-treatment (T0), post-treatment (T1), and at the 6 months follow-up (T2).

Response, remission, and recovery rates based on depression symptoms were evaluated. Response was considered as a 50% symptom reduction. Remission was defined when a score ≤ 7 was reached on the HamD. Recovery was defined when a remission at T1 was stable for at least 6 months (T2) (DGPPN et al., 2009, adapted: June 2015).

### Therapist Competence

All therapists were graduates of the MCT Institute^1^ diploma program which is a 128-h training curriculum in metacognitive therapy that includes supervision by accredited MCT supervisors. Therapists for both groups were the same. Adherence and competence were checked by monthly expert supervision.

### Statistics

Statistical analysis was performed using IBM SPSS (version 24). Categorical variables were compared using Chi-square tests. Group comparisons at the beginning of the study were analyzed using *t* tests. To determine depressive symptoms over the course of the study, mixed model ANOVA with HamD sum score as the dependent variable, group as between-subjects variable, and time at pre-treatment (T0), post-treatment (T1), and follow-up (T2) as the repeated measures factor was performed. If the assumption of sphericity was violated, the Greenhouse Geisser correction was applied. Analyses were performed using intention-to-treat principles and using last observation carried forward (LOCF) for missing data. Effect sizes were analyzed using Cohen’s *d* effect sizes.

## Results

Patients with MDD and PDD were similar concerning gender distribution (66.6 versus 53.3%), age (42.9 ± 13.2 versus 39.3 ± 8.4 years), partner status (53.3% partnered in both groups), and employment status (93.3 versus 80% employed) (for *p*, see Table 1). Patients with PDD had significantly more trials of former psychotherapy during the present episode (80 versus 0% in the MDD group), and all patients in the PDD group had at least one former trial with antidepressant medication, compared to none in the MDD group. Considering the mean HamD score at T0 (Table 2), severity of depression was similar in both groups before therapy started. In both groups, severity was moderate to severe.

**Table 2:** Means, standard deviations, effect sizes, and summary statistics for the primary and secondary outcome measures comparing cases of persistent depression vs. cases of current major depression [intention to treat analysis (*n* = 30)].

		Pre-treatment	Post-treatment	Follow-up	Effect size for T0-T1	Effect size for T0-T2	Interaction effect (time × group)	Main effect *F* (time)	Main effect *F* (group)
HamD	PDD	20.7 ± 5.7	5.1 ± 4.1	4.2 ± 3.9	−3.1	−3.4	*F*(1.1, 32) = 0.3, *p* = 0.62	*F*(1.1, 32) = 127.6, *p* < 0.01	*F*(1, 28) = 1.2, *p* = 0.28
	MDD	21.2 ± 4.6	7.3 ± 5.7	5.9 ± 6	−2.7	−2.9			
BAI	PDD	16.5 ± 11.6	9.5 ± 7.6	5.3 ± 3.8	−0.7	−1.3	*F*(1.4, 38.9) = 0.8, *p* = 0.41	*F*(1.4, 38.9) = 14.9, *p* < 0.01	*F*(1, 28) = 1.1, *p* = 0.31
	MDD	18.9 ± 13.3	10.4 ± 9	10.8 ± 10.2	−0.7	−0.7			
PBRS	PDD	20.6 ± 6.1	15.4 ± 7	12.4 ± 3.6	−0.8	−1.6	*F*(2, 56) = 1.9, *p* = 0.17	*F*(2, 56) = 18.4, *p* < 0.01	*F*(1, 28) = 0.9, *p* = 0.34
	MDD	22 ± 4.9	14.7 ± 5.3	16.5 ± 7.6	−1.4	−0.8			
NBRS	PDD	28.8 ± 6.7	23.4 ± 7.3	18.9 ± 5.1	−0.8	−1.7	*F*(1.6, 44.8) = 2.6, *p* = 0.1	*F*(1.6, 44.8) = 32.2, *p* < 0.01	*F*(1, 28) = 0.1, *p* = 0.94
	MDD	28.3 ± 6.9	21.5 ± 5.1	21.7 ± 4.8	−1.1	−1.1			
MCQ-30	PDD	62 ± 13	51.9 ± 13.8	44.8 ± 11.7	−0.8	−1.4	*F*(1.5, 42.3) = 1.1, *p* = 0.34	*F*(1.5, 42.3) = 27.6, *p* < 0.01	*F*(1, 28) = 1.6, *p* = 0.22
	MDD	67.5 ± 13.9	53.1 ± 12.1	52.5 ± 11.5	−1.1	−1.2			
RRS	PDD	50.7 ± 10.7	38.3 ± 11.5	32.2 ± 8.3	−1.1	−1.9	*F*(1.4, 37.9) = 1.4, *p* = 0.26	*F*(1.4, 37.9) = 33.2, *p* < 0.01	*F*(1, 28) = 1.6, *p* = 0.22
	MDD	53.3 ± 9.9	38.6 ± 9.5	39.5 ± 10.2	−1.5	−1.4			

*MDD, Current major depressive disorder; PDD, Persistent depressive disorder; HamD, Hamilton depression scale; BAI, Beck anxiety inventory; PBRS, Positive beliefs about rumination scale; NBRS, Negative beliefs about rumination scale; MCQ-30, Metacognition questionnaire; RRS, Ruminative response scale; d = 0.2–0.4: small effect; d = 0.5–0.7: medium effect; d ≥ 0.8: large effect*.

Comorbidity status was similar in both groups, with slightly more anxiety disorders in the MDD group, and slightly more personality disorders in the PDD group.

Depressive symptoms significantly improved in both groups as measured at T1 with HamD declining from 20.7 ± 5.7 to 5.1 ± 4.1 (*d* = −3.1) in PDD and from 21.2 ± 4.6 to 7.3 ± 5.7 (*d* = −2.7) in MDD. The mixed model ANOVA showed no statistically significant interaction effect between time and group (*F*(1.1,32) = 0.3, *p* = 0.62). Regardless of group, a significant main effect for time (*F*(1.1,32) = 127.6, *p* < 0.01) could be found. There was no statistically significant main effect for group, meaning that the groups did not differ significantly (*F*(1,28) = 1.2, *p* = 0.28).

With age as a covariate in a mixed model ANCOVA, there remained no statistically significant difference between the two groups (*F*(3,25) = 2.49, *p* = 0.083). A main effect for age could be found though (*F*(1,27) = 8, *p* < 0.05 at T1 and *F*(1,27) = 5.9, *p* < 0.05 at T2). After stratification by age, no interaction effect between time and age could be found (*F*(1.2, 32.2) = 1.5, *p* = 0.23). Comparing HamD scores and effect sizes in the stratified sample, it could be seen that the effect on symptom reduction was even stronger in the group of younger patients (HamD at T0: 20.7 ± 5.6, T1: 4.4 ± 4.2, T2: 3.3 ± 3.8, *d*(T0,T1) = −3.3, *d*(T0, T2) = −3.6) than the effect found in the group of older patients (HamD at T0: 21.2 ± 4.6, T1: 8.3 ± 5.3, T2: 7.1 ± 5.7, *d*(T0, T1) = −2.6, *d*(T0,T2) = −2.7).

In summary, all depressive symptoms improved significantly in all patients independent of type of depression and age.

Results of the secondary outcomes are presented in Table 2, demonstrating that both groups were similar in reduction of BAI sum scores, positive and negative beliefs about rumination scale score, and ruminative response scale scores.

The ANOVA of the intention to treat analysis yielded significant improvements in the pre-treatment, post-treatment, follow-up comparison on all variables (main effect time: BAI: *F*(1.4, 38.9) = 14.9, *p* < 0.01; PBRS: *F*(2, 56) = 18.4, *p* < 0.01; NBRS *F*(1.6, 44.8) = 32.2, *p* < 0.01; MCQ-30: *F*(1.5, 42.3) = 27.6, *p* < 0.01; RRS: *F*(1.4, 37.9) = 33.2, *p* < 0.01). As Table 2 shows, no interaction effects between the two groups could be found. None of the main effects for group were statistically significant. In brief, both groups improved to a similar degree on secondary outcome parameters over time.

In both groups, large effect sizes (Cohen’s *d*) were found evaluating the pre-treatment post-treatment (T0-T1)- and pre-treatment-follow-up (T0-T2)- comparison of the HamD scores (T0-T2: PDD: *d* = −3.4, MDD: *d* = −2.9). Looking at the secondary outcome parameters, large effect sizes can be reported analyzing the scores of the PBRS (T0-T2: PDD: *d* = −1.6, MDD: *d* = −0.8), NBRS (T0-T2: PDD: *d* = −1.7, MDD: *d* = −1.1), MCQ-30 (PDD: *d* = −1.4, MDD: *d* = −1.2) and RRS (T0-T2: PDD: *d* = −1.9, MDD: *d* = −1.4). On the BAI, a large effect size was observed in PDD (T0-T2: *d* = −1.3) compared to a medium effect size in the MDD group (T0-T2: *d* = −0.7).

Response, remission, and recovery rates are presented in Table 3. Improvement of depressive symptoms was slightly better in PDD (response: 80%, remission: 80%, recovery: 80%) than MDD (response: 73.3%, remission: 66.7%, recovery: 66.7%) assessed by clinician-rated HamD. In no patient symptoms deteriorated. Treatment gains were ongoing in both groups after 6-month follow-up (HamD at T2: PDD: 4.2 ± 3.9, *n* = 12; MDD: 5.9 ± 6, *n* = 8), although there was more missing data in the MDD group at T2.

**Table 3:** Remission, response, and recovery rates according to HamD sum scores in the intention to treat analysis (*n* = 30) using last observation carried forward for missing data.

		Response T2	Remission T2	Recovery
HamD	PDD (*n* = 15)	80% (12)	80% (12)	80% (12)
	MDD (*n* = 15)	73.3% (11)	66.7% (10)	66.7% (10)

*HamD, Hamilton depression scale; MDD, Major depressive disorder; PDD, Persistent depressive disorder*.

The number of MCT treatment sessions was only slightly different in each group (MDD: 15.5 ± 8.7 sessions versus 17.8 ± 7.5 sessions in the PDD group) (data not shown). Using Spearman’s rank-order correlation, we did not find a correlation between clinical improvement and number of MCT sessions (data not shown).

All patients improved concerning HamD sum scores. Considering single items of the HamD scale, both groups improved most (≥2 points difference between T0 and T1) on item 1 (“depressed mood”). PDD patients also improved markedly (>2 points) on item 7 (“work and interests”) and item 10 (“anxiety – psychic”). Concerning remaining items in remitted patients of both groups, no item could be identified as outstanding. All items in both groups of remitted patients had a mean score below 1 at the end of treatment (T1).

## Discussion

The main results of our study are that metacognitive therapy in patients with MDD and PDD was associated with significant improvement in terms of HamD sum score, response and remission rates at post-treatment and at 6 months after the end of treatment. Of particular interest, effect sizes and clinical outcomes were broadly comparable across patients with PDD and MDD. A slight difference can be seen in the level of recovery where we observed an almost 15% difference favoring those with PDD. While caution should be exercised in interpreting these data, they do suggest that MCT is associated with large improvements in both groups of patients. Furthermore, our results are encouraging in that patients with high levels of non-response to previous treatment may profit from MCT. Our results are not biased by different dosages of MCT as documented by a similar mean rate of MCT sessions in both groups. A further interesting aspect is that our data indicate that the effect of MCT is independent of patient’s age. Effect sizes were higher in younger patients, but still large in older patients, meaning that possible differences in depressive symptoms relating to age do not hinder the therapy progress.

Our data are in accordance with other studies demonstrating strong efficacy of MCT in depression. In recent meta-analyses, large controlled effect sizes were reported favoring MCT over wait-list control and CBT in depression and anxiety disorders (Normann et al., 2014; Normann and Morina, 2018). A recent randomized study with 48 depressed participants from New Zealand compared MCT to CBT and reported a similar reduction in depressive symptoms with both treatments (Jordan et al., 2014), but therapists were not trained in MCT. Effect sizes were *d* = 0.96 in the MCT group and *d* = 0.60 in the CBT group at week 4. In an analysis of the effects of these treatments on executive functioning, MCT appeared to produce superior outcomes than CBT (Groves et al., 2015). A three-armed depression study from Iran including 10 patients in a MCT group, 10 patients in a CBT group, and 13 patients in a comparison group with pharmacological treatment equally showed similar outcomes in MCT and CBT (Ashouri et al., 2013), but again therapists were not trained in MCT. Case series studies from England and one from Denmark showed significant improvements in depressive symptoms, rumination, and metacognitive beliefs after MCT (Wells et al., 2012; Callesen et al., 2014).

The novel aspect of the current study is the comparison of effects in patients with current MDD against those with persistent MDD. To date, two published studies have examined the effects of MCT in chronic and persistent depression (Wells et al., 2012; Papageorgiou and Wells, 2015) with results that are comparable with those in the current study. However, these uncontrolled earlier studies did not directly compare the effects in MDD with those in PDD.

In the current study, we observed that in both treated cohorts large and significant improvements in underlying thinking processes (rumination) measured, e.g., by the RRS and metacognitive beliefs were shown. The levels of improvement were similar in each case, which suggests that persistent depression is not associated with a lower level of change in hypothesized underlying causal mechanisms in patients undergoing MCT. This raises the question of why the PDD group had failed previous treatment attempts with antidepressant medication or CBT. One possibility is that the earlier treatments did not directly modify metacognitive beliefs and reduce the extent of rumination. In fact, it is possible that failed treatment attempts may strengthen unhelpful metacognitive beliefs about the uncontrollability of depressive thinking such that patients with PDD are more likely to search for a solution to their depression that relies less on using their own executive control processes to overcome the problem. This could contribute to a persistence of rumination and maladaptive thinking patterns.

Moreover, the study addresses the question of distinguishing between different types of depression becomes less relevant when applying MCT. MCT modifies underlying processes of depression that are not explicitly targeted by CBT or other psychotherapy methods. Changing the style with which a person deals with cognitions and modifying metacognitive beliefs may be more important in beating depression than dealing with the content of negative automatic thoughts or schemas, but this hypothesis needs to be investigated further.

A recent study by Timm et al. (2017) demonstrated that changes in repetitive negative thinking are important not only on a trait level (macro-level), but also on a micro-level of moment-to-moment experiencing during daily life. They found that both trait and state processes of affective and cognitive processes impact the longer course of major depression (Timm et al., 2017). An important question arises whether MCT effectively changes cognitions on both levels, and whether this may result in long-lasting effects for prevention of further depression. According to our results, positive and negative beliefs about rumination and rumination itself changed significantly in both patient groups, accompanied by lasting remission after 6 months follow-up.

Interestingly, we did not find an association between number of MCT sessions and clinical improvement, which may suggest that in some patients, fewer MCT sessions may be sufficient for therapeutic success. This effect may also underlie the relatively large standard deviation in the number of MCT sessions in both groups, since reaching therapeutic goals was a criterion to end treatment.

Since we did not find an outstanding remaining HamD item in remitted patients of both groups, one may conclude that MCT has a global effect on all dimensions of MDD or PDD, respectively. In summary, depressive symptoms as assessed by expert rating (HamD) decreased in both study groups during MCT with high effect sizes. Dysfunctional metacognition (PBRS, NBRS, and MCQ-30) decreased with high effect sizes as well as the style of responding to rumination (RRS). Gender, age, and family status had no effect on the treatment outcome as assessed by multiple measurements ANOVA with the respective variables as confounders.

## Limitations

Due to the small sample size and a missing independent control group, the study does not prove efficacy of the treatment. We cannot control for non-specific factors such as the effect of the therapeutic alliance and also for factors such as the passage of time and repeated testing. We therefore have no information on possible improvements without any intervention. The HamD rating was not completed by independent raters, but by therapists involved in the study. Although it was not necessarily the therapist who did the therapy with the assessed person, this may have resulted in an overestimation of change within therapy. Also inter-rater reliability was not measured and may limit the results. In addition, the Hamilton rating scale for depression itself may count as a limitation. Even though it is one of the most commonly used measures for depression, some of its quality criteria are poor (Bagby et al., 2004). Furthermore, the standardized post-evaluation of diagnosis is missing due to the naturalistic design. One further limitation has to be kept in mind, we analyzed data using the LOCF procedure, which may influence effect sizes. The use of LOCF can be criticized (Lachin, 2016) as it may overestimate or underestimate treatment effects. However, in our study, we consider this a conservative method that would most likely cause differences between the two groups (our null hypothesis) when one of the groups is considered more treatment resistant. In practice, there were few missing values and so the impact is in any case likely to be small.

## Conclusions

Independent of the subtype of depression, metacognitive therapy was associated with significant improvement of depressive symptoms, symptoms of comorbid disorders, and changes in thinking styles and metacognitions. This indicates that the underlying processes of persistent depression and current depression may be equally modifiable by MCT irrespective of whether or not depression is considered treatment-refractory.

## Data Availability

The datasets generated for this study are available on request to the corresponding author.

## Ethics Statement

This study was carried out in accordance with the recommendations of the ethics committee of the Hannover Medical School with written informed consent from all subjects. All subjects gave written informed consent in accordance with the Declaration of Helsinki. The protocol was approved by the ethics committee of the Hannover Medical School.

## Author Contributions

LW, US, and KK designed the original concept. JG and JN recruited the patients. AW trained the therapists in MCT. LW, JG, and JN carried out the assessments and therapies. LW, AW, US, and KK conducted the data analysis and wrote the paper.

### Conflict of Interest Statement

The authors declare that the research was conducted in the absence of any commercial or financial relationships that could be construed as a potential conflict of interest.

## Footnotes

^1^www.mct-institute.com

## References

[ref1] Ashouri A. Atef Vahid M. K. Gharaee B. Rasoulian M. (2013). Effectiveness of meta-cognitive and cognitive-behavioral therapy in patients with major depressive disorder. Iran. J. Psychiatry Behav. Sci. 7, 24–34. http://www.ncbi.nlm.nih.gov/pubmed/24644507 PMC3939994 24644507

[ref2] Bagby R. M. Ryder A. G. Schuller D. R. Marshall M. B. (2004). The Hamilton depression rating scale: has the gold standard become a lead weight? Am. J. Psychiatry 161, 2163–2177. 10.1176/appi.ajp.161.12.2163 15569884

[ref3] Bevan D. Wittkowski A. Wells A. (2013). A multiple-baseline study of the effects associated with metacognitive therapy in postpartum depression. J. Midwifery Womens Health 58, 69–75. 10.1111/j.1542-2011.2012.00255.x 23374492

[ref4] Callesen P. Jensen A. B. Wells A. (2014). Metacognitive therapy in recurrent depression: a case replication series in Denmark. Scand. J. Psychol. 55, 60–64. 10.1111/sjop.12089 24256292

[ref5] CIPS-Collegium Internationale Psychiatriae Scalarum (2015). Internationale Skalen für Psychiatrie. Göttingen: Hogrefe.

[ref6] Dammen T. Papageorgiou C. Wells A. (2015). An open trial of group metacognitive therapy for depression in Norway. Nord. J. Psychiatry 69, 126–131. 10.3109/08039488.2014.936502 25124119

[ref7] DGPPN BÄK, KBV, AWMF, AkdÄ, BPtK, BApK, DAGSHG, DEGAM, DGPM, DGPs, DGRW (2009, adapted: June 2015). S3-Leitlinie/Nationale VersorungsLeitlinie Unipolare Depression - Langfassung.

[ref8] Drost J. van der Does W. van Hemert A. M. Penninx B. W. Spinhoven P. (2014). Repetitive negative thinking as a transdiagnostic factor in depression and anxiety: a conceptual replication. Behav. Res. Ther. 63, 177–183. 10.1016/j.brat.2014.06.004, PMID: 25461794

[ref9] Farahmand V. Hassanzadeh R. Mirzaian B. Fayyazi Bordbar M. R. Feizi J. (2014). The efficacy of group metacognitive therapy on self-esteem and mental health of patients suffering from major depressive disorder. Iran. J. Psychiatry Behav. Sci. 8, 4–10. http://www.ncbi.nlm.nih.gov/pubmed/25053952 25053952 PMC4105599

[ref10] Gan P. Xie Y. Duan W. Deng Q. Yu X. (2015). Rumination and loneliness independently predict six-month later depression symptoms among Chinese elderly in nursing homes. PLoS One 109:e0137176. 10.1371/journal.pone.0137176 PMC4559426 26334298

[ref11] Groves S. J. Porter R. J. Jordan J. Knight R. Carter J. D. McIntosh V. V. (2015). Changes in neuropsychological function after treatment with metacognitive therapy or cognitive behavior therapy for depression. Depress. Anxiety 32, 437–444. 10.1002/da.22341 25677736

[ref12] Hagen R. Hjemdal O. Solem S. Kennair L. E. O. Nordahl H. M. Fisher P.. (2017). Metacognitive therapy for depression in adults: a waiting list randomized controlled trial with six months follow-up. Front. Psychol. 8:31. 10.3389/fpsyg.2017.00031, PMID: 28174547 PMC5258745

[ref13] Hardeveld F. Spijker J. De Graaf R. Nolen W. A. Beekman A. T. (2010). Prevalence and predictors of recurrence of major depressive disorder in the adult population. Acta Psychiatr. Scand. 122, 184–191. 10.1111/j.1600-0447.2009.01519.x 20003092

[ref14] Hardeveld F. Spijker J. De Graaf R. Nolen W. A. Beekman A. T. (2013). Recurrence of major depressive disorder and its predictors in the general population: results from the Netherlands Mental Health Survey and Incidence Study (NEMESIS). Psychol. Med. 43, 39–48. 10.1017/s0033291712002395 23111147

[ref15] Hollon S. D. DeRubeis R. J. Fawcett J. Amsterdam J. D. Shelton R. C. Zajecka J. (2014). Effect of cognitive therapy with antidepressant medications vs antidepressants alone on the rate of recovery in major depressive disorder: a randomized clinical trial. JAMA Psychiat. 71, 1157–1164. 10.1001/jamapsychiatry.2014.1054 PMC4315327 25142196

[ref16] Jordan J. Carter J. D. McIntosh V. V. Fernando K. Frampton C. M. Porter R. J. (2014). Metacognitive therapy versus cognitive behavioural therapy for depression: a randomized pilot study. Aust. N. Z. J. Psychiatry 48, 932–943. 10.1177/0004867414533015 24810871

[ref17] Kabacoff R. I. Segal D. L. Hersen M. Van Hasselt V. B. (1997). Psychometric properties and diagnostic utility of the Beck anxiety inventory and the state-trait anxiety inventory with older adult psychiatric outpatients. J. Anxiety Disord. 11, 33–47. 10.1016/s0887-6185(96)00033-3 9131880

[ref18] Klein D. N. (2010). Chronic depression: diagnosis and classification. Curr. Dir. Psychol. Sci. 19, 96–100. 10.1177/0963721410366007

[ref19] Kriston L. von Wolff A. Westphal A. Holzel L. P. Harter M. (2014). Efficacy and acceptability of acute treatments for persistent depressive disorder: a network meta-analysis. Depress. Anxiety 31, 621–630. 10.1002/da.22236 24448972

[ref20] Kuehner C. Weber I. (1999). Responses to depression in unipolar depressed patients: an investigation of Nolen-Hoeksema’s response styles theory. Psychol. Med. 29, 1323–1333. 10616938 10.1017/s0033291799001282

[ref21] Lachin J. M. (2016). Fallacies of last observation carried forward analyses. Clin. Trials 13, 161–168. 10.1177/1740774515602688 26400875 PMC4785044

[ref22] Lyubomirsky S. Layous K. Chancellor J. Nelson S. K. (2015). Thinking about rumination: the scholarly contributions and intellectual legacy of Susan Nolen-Hoeksema. Annu. Rev. Clin. Psychol. 11, 1–22. 10.1146/annurev-clinpsy-032814-112733, PMID: 25581241

[ref23] Nolen-Hoeksema S. Morrow J. (1991). A prospective study of depression and posttraumatic stress symptoms after a natural disaster: the 1989 Loma Prieta earthquake. J. Pers. Soc. Psychol. 61, 115–121. 1890582 10.1037//0022-3514.61.1.115

[ref24] Nolen-Hoeksema S. Wisco B. E. Lyubomirsky S. (2008). Rethinking rumination. Perspect. Psychol. Sci. 3, 400–424. 10.1111/j.1745-6924.2008.00088.x 26158958

[ref25] Normann N. Morina N. (2018). The efficacy of metacognitive therapy: a systematic review and meta-analysis. Front. Psychol. 9:2211. 10.3389/fpsyg.2018.02211, PMID: 30487770 PMC6246690

[ref26] Normann N. van Emmerik A. A. Morina N. (2014). The efficacy of metacognitive therapy for anxiety and depression: a meta-analytic review. Depress. Anxiety 31, 402–411. 10.1002/da.22273 24756930

[ref27] Papageorgiou C. Wells A. (2001a). Positive beliefs about depressive rumination: development and preliminary validation of a self-report scale. Behav. Ther. 32, 13–26. 10.1016/S0005-7894(01)80041-1

[ref28] Papageorgiou C. Wells A. (2001b). Metacognitive beliefs about rumination in recurrent major depression. Cogn. Behav. Pract. 8, 160–163. 10.1016/S1077-7229(01)80021-3

[ref29] Papageorgiou C. Wells A. (2003). An empirical test of a clinical metacognitive model of rumination and depression. Cogn. Ther. Res. 27, 261–273. 10.1023/A:1023962332399

[ref30] Papageorgiou C. Wells A. (2015). Group metacognitivetherapy for severe antidepressant and CBT resistant depression: a baseline-controlled trial. Cogn. Ther. Res. 39, 14–22. 10.1007/s10608-014-9632-x

[ref31] Penninx B. W. Nolen W. A. Lamers F. Zitman F. G. Smit J. H. Spinhoven P. (2011). Two-year course of depressive and anxiety disorders: results from the Netherlands Study of Depression and Anxiety (NESDA). J. Affect. Disord. 133, 76–85. 10.1016/j.jad.2011.03.027 21496929

[ref32] Riihimaki K. A. Vuorilehto M. S. Melartin T. K. Isometsa E. T. (2014). Five-year outcome of major depressive disorder in primary health care. Psychol. Med. 44, 1369–1379. 10.1017/s0033291711002303 22085687

[ref33] Seemuller F. Meier S. Obermeier M. Musil R. Bauer M. Adli M. (2014). Three-year long-term outcome of 458 naturalistically treated inpatients with major depressive episode: severe relapse rates and risk factors. Eur. Arch. Psychiatry Clin. Neurosci. 264, 567–575. 10.1007/s00406-014-0495-7 24590257

[ref34] Timm C. Ubl B. Zamoscik V. Ebner-Priemer U. Reinhard I. Huffziger S. (2017). Cognitive and affective trait and state factors influencing the long-term symptom course in remitted depressed patients. PLoS One 12:e0178759. 10.1371/journal.pone.0178759 28575049 PMC5456349

[ref35] Wells A. (2000). Emotional disorders and metacognition: Innovative cognitive therapy. Chichester UK: Wiley.

[ref36] Wells A. (2009). Metacognitive therapy for anxiety and depression. New York: Guilford Press.

[ref37] Wells A. (2011). Metakognitive Therapie bei Angststörungen und Depression. Weinheim: Beltz.

[ref38] Wells A. Cartwright-Hatton S. (2004). A short form of the metacognitions questionnaire: properties of the MCQ-30. Behav. Res. Ther. 42, 385–396. 10.1016/s0005-7967(03)00147-5 14998733

[ref39] Wells A. Fisher P. Myers S. Wheatley J. Patel T. Brewin C. R. (2012). Metacognitive therapy in treatment-resistant depression: a platform trial. Behav. Res. Ther. 50, 367–373. 10.1016/j.brat.2012.02.004 22498310

[ref40] Wells A. Matthews G. (1994). Attention and emotion a clinical perspective. Hove, UK: Lawrence Erlbaum Associates.

[ref41] Whiteford H. A. Degenhardt L. Rehm J. Baxter A. J. Ferrari A. J. Erskine H. E. (2013). Global burden of disease attributable to mental and substance use disorders: findings from the Global Burden of Disease Study 2010. Lancet 382, 1575–1586. 10.1016/s0140-6736(13)61611-6 23993280

[ref43] Wiersma J. E. van Oppen P. van Schaik D. J. van der Does A. J. Beekman A. T. Penninx B. W. (2011). Psychological characteristics of chronic depression: a longitudinal cohort study. J. Clin. Psychiatry 72, 288–294. 10.4088/jcp.09m05735blu 21450151

[ref44] Wittchen H. Zaudig M. Fydrich T. (1997). “SKID-I und SKID-II” in Strukturiertes Klinisches Interview für DSM-IV (Göttingen: Hogrefe).

[ref45] Yilmaz A. E. Gencoz T. Wells A. (2015). Unique contributions of metacognition and cognition to depressive symptoms. J. Gen. Psychol. 142, 23–33. 10.1080/00221309.2014.964658 25539184

